# Successful treatment with pembrolizumab for microsatellite instability-high thymic carcinoma: A case report

**DOI:** 10.1016/j.rmcr.2025.102272

**Published:** 2025-07-23

**Authors:** Yuichi Sakamori, Kentaro Hamada, Hiroaki Kawachi, Mako Yamoto, Akari Fukao, Satoshi Terashita, Kizuku Watanabe, Tatsuyoshi Ikeue, Takakazu Sugita

**Affiliations:** Department of Respiratory Medicine, Japan Red Cross Society Wakayama Medical Center, 4-20 Komatsubara-dori, Wakayama City, 640-8558, Japan

**Keywords:** Thymic carcinoma, MSI-High, Pembrolizumab, Immunotherapy

## Abstract

**Introduction:**

Thymic carcinoma is a rare, aggressive malignancy, and in cases of metastasis, palliative chemotherapy is the typical treatment. Microsatellite instability (MSI)-high tumors are more likely to respond to immune checkpoint inhibitors, such as pembrolizumab, than to other types of chemotherapy. However, MSI-high thymic carcinoma is extremely rare, with limited reports on the efficacy of pembrolizumab in this context.

**Case:**

A 72-year-old woman with metastatic thymic carcinoma (Masaoka stage IVA) and concurrent breast cancer was confirmed to have an MSI-high status through biopsy. Following progression after first-line carboplatin and paclitaxel, pembrolizumab (400 mg) was administered as fourth-line therapy. CT imaging after one month revealed significant tumor shrinkage, including lesions outside the irradiation field. A sustained partial response was observed for four months after a single cycle of pembrolizumab.

**Discussion:**

This case demonstrates the potential efficacy of pembrolizumab in treating MSI-high thymic carcinoma, emphasizing the importance of MSI testing in such cases. Routine MSI evaluation should be considered to identify candidates for immunotherapy. Further research is needed to confirm the role of pembrolizumab in this rare malignancy and to establish its broader applicability under similar conditions.

## 1 Introduction

Thymic carcinomas are a rare subset of thymic epithelial tumors characterized by their aggressive behavior compared with other thymic tumors. The global incidence of thymic carcinomas is estimated to be 1.3–3.2 cases per million people [1,2], with a low 5-year survival rate of 30–50 % [3]. Pathological risk factors remain unknown. For metastatic thymic carcinoma, treatment options are limited to palliative chemotherapy or supportive care. Given the rarity of this disease, there is no standard established chemotherapy regimen. Platinum-based chemotherapy, with or without anthracycline, is commonly used as first-line therapy, with response rates ranging from 20 to 50 % [4]. Unfortunately, no survival benefit has been demonstrated for second-line treatments. The NCCN guidelines recommend single-agent cytotoxic or molecularly targeted therapies for refractory cases [5], (2024, https://www.nccn.org/professionals/physician_gls/pdf/thymic.pdf). In phase II trials in the United States and Europe, Pembrolizumab has shown effects, particularly when PD-L1 expression (IHC 22C3) is high [6,7]. However, routine testing for PD-L1 expression and the use of pembrolizumab on the basis of these results have not been approved for the treatment of thymic carcinoma. However, microsatellite instability (MSI)-high tumors are more likely to respond to anti-PD-1 antibodies, and pembrolizumab and nivolumab are approved for MSI-high solid tumors; however, MSI-high thymic carcinomas are exceedingly rare, with no reported cases treated with pembrolizumab.

Here, we report the first case of thymic carcinoma with elevated MSI to be successfully treated with pembrolizumab.

## 2 Case

A 72-year-old woman presented to the Japanese Red Cross Wakayama Medical Center in August 2023 with a left breast mass and an anterior mediastinal mass. She had no history of smoking and no significant medical history, except for chemotherapy for right breast cancer 24 years earlier.

**Fig. 1:**
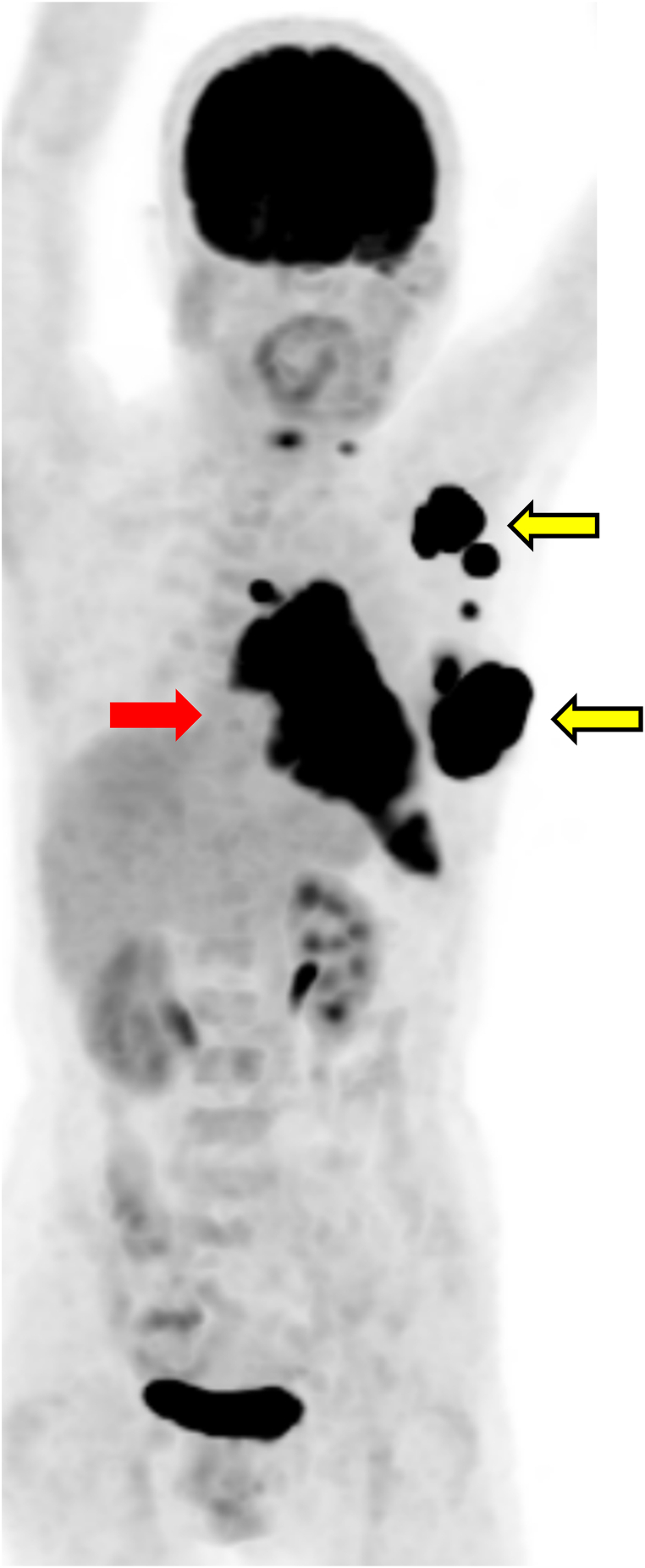
Positron emission tomography. Before treatment, high 18F-fluorodeoxyglucose accumulation was observed in the anterior mediastinal and pleural lesions (red arrows), which were thought to be thymic carcinomas, and in the left breast and axillary lymph nodes (yellow arrows), which were thought to be breast cancer.

**Fig. 2:**
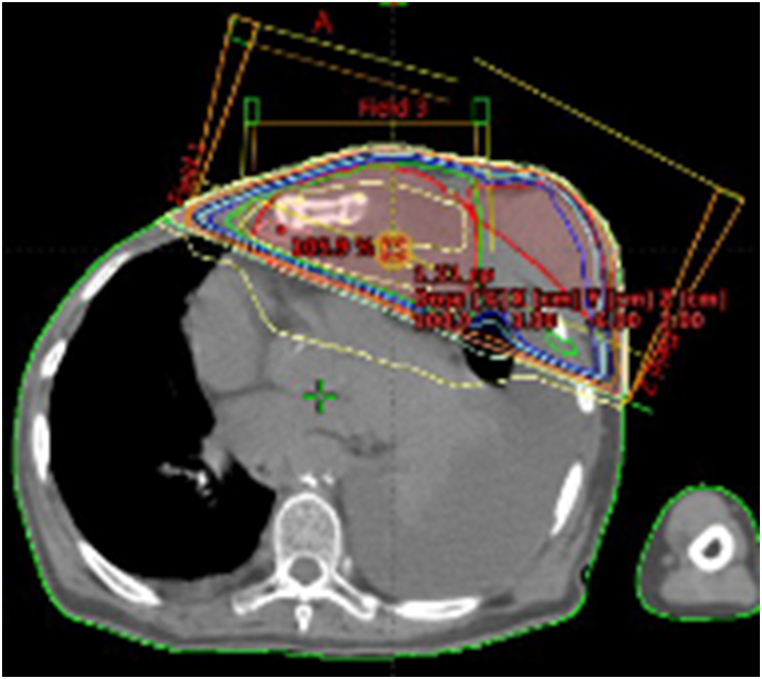
Schematic of the CT simulation of radiation. The anterior longitudinal part of the thymic carcinoma and the left breast cancer were irradiated with 30 Gy/15 Fr of palliative irradiation. No attempt was made to irradiate the disseminated thymic carcinoma lesion in the cardiac diaphragm.

Biopsy of the left breast confirmed invasive carcinoma (ER+, PgR+, HER2 1+, Ki-67 50 %), whereas biopsy of the anterior mediastinal mass revealed squamous cell thymic carcinoma (CD5 positive). CT imaging revealed a left breast mass, enlarged axillary lymph nodes, and a mass with pleural effusion extending from the anterior mediastinum to the left cardiac diaphragm. PET-CT revealed 18F-FDG uptake in pleural lesions (red arrow) consistent with thymic carcinoma, as well as in the left breast and axillary lymph nodes (yellow arrow), consistent with breast cancer (Fig. 1). On the basis of these findings, the patient was diagnosed with Masaoka stage IVA thymic carcinoma and breast cancer. First-line treatment with carboplatin (AUC6) and paclitaxel (200 mg/m^2^) was initiated in September 2023. The disease progressed, and subsequent second-line therapy with lenvatinib and third-line therapy with S-1 (tegafur, gimeracil, and oteracil potassium) were also ineffective. However, by July 2024, imaging revealed progression in both lesions. Palliative irradiation (45 Gy/15 fractions) was performed on the breast and anterior mediastinal lesions (Fig. 2).

**Fig. 3:**
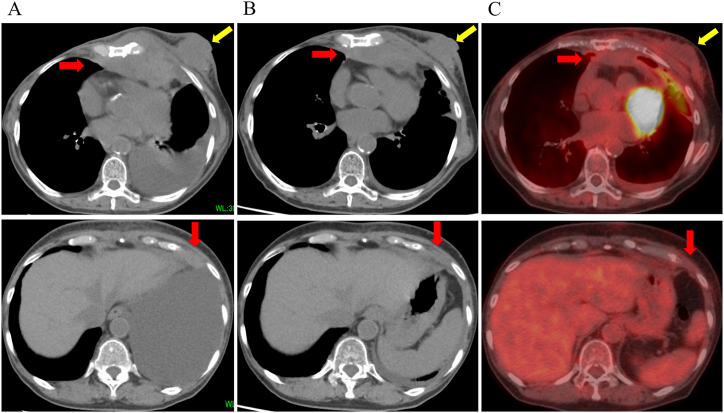
Temporal changes in tumor lesions on CT images. (A) A growing thymic carcinoma in the anterior mediastinum (red arrow), a disseminated lesion of thymic carcinoma in the cardiac diaphragm angle (red arrow) and a growing breast carcinoma in the left breast (yellow arrow) are shown. (B) Two months after irradiation and pembrolizumab administration, the lesions tended to shrink, and the disseminated lesion, which was outside of the irradiation range, also showed shrinkage. (C) Four months after the start of treatment, the lesions in the anterior longitudinal cage and left breast and the disseminated lesions were further reduced. CT, computed tomograph.

To evaluate the MSI status, a biopsy sample from 2022 was tested by means of an MSI-IVD kit (FALCO Biosystems). Although the patient's family history did not meet the criteria for Lynch syndrome per the Amsterdam II or revised Bethesda guidelines (7,8), the tumor was confirmed to be MSI-high. In August 2024, pembrolizumab (400 mg) was administered as fourth-line therapy. One month later, CT imaging in September 2024 revealed a significant reduction in the anterior mediastinal tumor (red arrow) and left breast mass (yellow arrow). Additionally, the lesion at the cardiac diaphragm, which was outside the irradiation field, showed shrinkage. PET imaging in December 2024 confirmed a partial response in the thymic carcinoma (Fig. 3). Throughout the treatment period, no adverse effects were observed.

## 3 Discussion

This case highlights the successful use of pembrolizumab in the treatment of MSI-high thymic carcinoma, an exceptionally rare condition. In prior studies, including phase II clinical trials [6,7], the efficacy of pembrolizumab was demonstrated in advanced thymic carcinoma, particularly in tumors with high PD-L1 expression. In a single-arm phase II trial, pembrolizumab achieved a response rate of 22.5 % and a median progression-free survival of 4.2 months in patients with PD-L1 expression ≥50 % [6]. Another phase II trial in Korea reported a partial response rate of 28.6 % in thymoma patients [7]. These findings suggest that pembrolizumab may be a promising treatment option for refractory thymic carcinoma that has progressed after first-line therapy.

However, pembrolizumab is not currently approved for the treatment of thymic carcinoma without biomarker testing. The MSI-high status or high tumor mutational burden (TMB) must be confirmed before considering immune checkpoint inhibitors. MSI-high tumors are well-established biomarkers for predicting the efficacy of immune checkpoint inhibitors [8]. Pembrolizumab has been shown to be effective in MSI-high/dMMR noncolorectal cancers, with a 34.3 % response rate, median progression-free survival of 4.1 months, and median overall survival of 23.5 months [8]. Despite these promising results, MSI-high thymic carcinoma is exceedingly rare, with an estimated prevalence of only 2.3–3.8 % [9,10].

To our knowledge, this is the first reported case of metastatic thymic carcinoma with confirmed MSI-high status demonstrating a sustained response to pembrolizumab. Even more remarkable is the sustained response observed four months after the discontinuation of a single cycle of pembrolizumab. These findings suggest that the MSI-high status of thymic carcinoma may represent a unique subset of patients who could benefit significantly from immune checkpoint inhibitors.

Considering these findings, routine MSI evaluation should be incorporated into the management of thymic carcinoma to identify potential candidates for immunotherapy. Further studies are warranted to better understand the role of pembrolizumab and other immune checkpoint inhibitors in this rare malignancy. This case highlights several important clinical implications. First, it demonstrates that pembrolizumab can be effective against MSI-high thymic carcinoma, a rare but potentially immunogenic tumor type. Given the limited treatment options for advanced thymic carcinoma, MSI testing may help identify patients who could benefit from immune checkpoint inhibitors. Notably, the sustained partial response following only a single administration of pembrolizumab suggests that even short-term exposure to immunotherapy may induce meaningful and durable responses in certain MSI-high tumors. Therefore, routine MSI evaluation should be considered in the diagnostic workup for thymic carcinoma.

Nevertheless, this report has some limitations. As a single case study, the generalizability of the findings is inherently limited. In this case, radiotherapy was administered to both the anterior mediastinal tumor and the left breast lesion prior to pembrolizumab treatment, and both lesions showed marked shrinkage after therapy. Among the multiple lesions, only the lesion at the pericardiac-diaphragmatic angle—indicated by the red arrow in Fig. 3—was located outside the irradiation field. Therefore, this was the only site where the isolated effect of pembrolizumab could be objectively assessed. Given that most other lesions were within the irradiated areas, the overall therapeutic response may be confounded by prior radiation, and the interpretation of pembrolizumab's efficacy should be made with caution. In addition, the follow-up period was relatively short, and long-term outcomes such as overall survival and time to progression remain unknown.

## 4 Conclusions

This case represents the first reported instance of MSI-high thymic carcinoma successfully treated with pembrolizumab, demonstrating the potential efficacy of immune checkpoint inhibitors in this rare malignancy. The observed sustained response, even after a single cycle of pembrolizumab, highlights the significance of the MSI-high status as a potential biomarker for predicting treatment outcomes in thymic carcinoma patients.

Our findings underscore the importance of incorporating routine MSI testing into the management of metastatic thymic carcinoma to identify candidates for immunotherapy. Further clinical studies are warranted to confirm the role of pembrolizumab and other immune checkpoint inhibitors in MSI-high thymic carcinoma and to explore their broader applicability in this rare disease.

## CRediT authorship contribution statement

**Yuichi Sakamori:** Writing – original draft, Visualization, Software, Resources, Methodology, Investigation, Data curation, Conceptualization. **Kentaro Hamada:** Writing – review & editing, Resources. **Hiroaki Kawachi:** Writing – review & editing. **Mako Yamoto:** Writing – review & editing. **Akari Fukao:** Writing – review & editing. **Satoshi Terashita:** Writing – review & editing. **Kizuku Watanabe:** Writing – review & editing. **Tatsuyoshi Ikeue:** Writing – review & editing. **Takakazu Sugita:** Writing – review & editing.

## Informed consent

Oral informed consent was obtained from the patient for publication of this case report and accompanying images. A copy of the written consent form is available for review by the editorial office of this journal.

## Ethical approval

The authors are accountable for all aspects of the work in ensuring that questions related to the accuracy or integrity of any part of the work are appropriately investigated and resolved. All procedures performed in this study were in accordance with the ethical standards of the institutional and national research committees and with the Helsinki Declaration (as revised in 2013).

## Funding

None.

## Declaration of competing interest

The authors declare that they have no known competing financial interests or personal relationships that could have appeared to influence the work reported in this paper.

## Data Availability

The data generated in the present study are included in the figures and/or tables of this article.
